# Variation in the *Dicer* and *RAN* Genes Are Associated with Survival in Patients with Hepatocellular Carcinoma

**DOI:** 10.1371/journal.pone.0162279

**Published:** 2016-09-09

**Authors:** Mi Na Kim, Jung Oh Kim, Seung Min Lee, Hana Park, Ju Ho Lee, Kyu Sung Rim, Seong Gyu Hwang, Nam Keun Kim

**Affiliations:** 1 Department of Internal Medicine, CHA Bundang Medical Center, CHA University, Seongnam, South Korea; 2 Institute for Clinical Research, CHA Bundang Medical Center, CHA University, Seongnam, South Korea; 3 Department of Biomedical Science, College of Life Science, CHA University, Seongnam, South Korea; Yonsei University College of Medicine, REPUBLIC OF KOREA

## Abstract

Single-nucleotide polymorphisms (SNPs) in microRNA machinery genes might affect microRNA processing and subsequently impact tumorigenesis. The aim of this study was to investigate the associations between SNPs in microRNA machinery genes and hepatocellular carcinoma (HCC) in a Korean population. Genotyping of six SNPs in microRNA machinery genes was performed using blood samples from 147 patients with HCC and 209 healthy control subjects. None of the six SNPs in microRNA machinery genes were significantly associated with HCC development. However, among the models for six polymorphic loci—*DICER* (rs3742330 and rs13078), *DROSHA* (rs10719 and rs6877842), *RAN* (rs14035) and *XPO5* (rs11077)—one allele combination (A-A-T-C-C-C) showed synergistic effects in terms of an increased risk of HCC development (odds ratio = 8.881, 95% confidence interval [CI] = 1.889–41.750; *P* = 0.002). Multivariate Cox proportional hazard regression analysis showed a significant survival benefit for the *DICER* rs3742330 GG compared with the AA type (hazard ratio [HR], 0.314; 95% CI, 0.135–0.730; *P* = 0.007) and for the *RAN* rs14035 CT compared with the CC genotype (HR, 0.587; 95% CI, 0.349–0.987; *P* = 0.044). Although we found no direct association between *DICER* (rs3742330 and rs13078), *DROSHA* (rs10719 and rs6877842), *RAN* (rs14035) or *XPO5* (rs11077) polymorphisms and HCC risk, we demonstrated that *DICER* (rs3742330) and *RAN* (rs14035) were associated with the survival of HCC patients. Future studies with larger samples are needed to determine associations of SNPs in microRNA machinery genes with HCC risk and prognosis.

## Introduction

Hepatocellular carcinoma (HCC) is the fifth most common cancer and the third leading cause of cancer-related deaths worldwide. [1] HCC is caused by hepatitis B and C viruses, smoking, alcohol consumption, chemical exposure, aflatoxin B1, and intrinsic factors such as genetic mutations. [2] However, the mechanisms of how these risk factors affect the susceptibility and severity of HCC remains unclear.

MicroRNAs are short, non-coding RNAs of approximately ~23 nucleotides that act as post-transcriptional regulators of gene expression and have been implicated in the initiation and progression of various cancers. [3] In microRNA processing, DROSHA and its cofactor, DGCR8 process primary microRNAs into precursor-microRNAs (pre-microRNAs). Pre-microRNAs are then exported into the cytoplasm by the XPO5/Ran-GTP complex and further processed by a protein complex that includes DICER, TRBP, AGO1, and AGO2, leading to the production of mature microRNAs. [4,5]

Several studies have demonstrated the association between key genes of the microRNA biosynthetic pathway and the development of various cancer, including HCC. [6–9] For example, microRNA machinery genes including *DICER*, *DGCR8*, *AGO3*, and *AGO4* were found to be dysregulated in HCC. [10] Altered expression of DICER was associated with the development of lung and prostate cancers. [11–13] Taken together, these emerging lines of evidence suggest that microRNA machinery genes play important roles in cancer development and progression.

Single nucleotide polymorphisms (SNPs) have been widely implicated in cancer development and the response to treatmen. [14,15] The important role played by microRNAs in cancer [16] suggests that SNPs in microRNA machinery genes might affect microRNA processing and subsequently impact tumorigenesis. Recent studies have demonstrated an association between SNPs in microRNA machinery genes and the risk of several cancers through affecting the mature process of microRNAs. [16] However, to date, few studies have investigated the association between SNPs in microRNA machinery genes and HCC development and survival. Thus, in the present study, we determined whether polymorphisms in *DICER* (rs3742330 and rs13078), *DROSHA* (rs10719 and rs6877842), *RAN* (rs14035) and *XPO5* (rs11077) were associated with HCC development and survival in a Korean population.

## Materials and Methods

### Study population

A total of 147 cases with HCC diagnosed at CHA Bundang Medical Center from June 1996 to August 2008 were enrolled. The control group consisted of 229 individuals randomly selected from participants in a health-screening program. The clinical stage of HCC was evaluated on the basis of the TNM classification stage system. The patients were classified according to Child-Pugh classes as A, B, or C. In the survival analysis, one patient with HCC was excluded due to loss to follow-up. The Institutional Review Board of CHA Bundang Medical Center approved the present study, and written informed consent was obtained from all patients and control subjects.

### Analysis of microRNA machinery gene polymorphisms

Genomic DNA was extracted from peripheral blood samples collected with an anticoagulant using a G-DEX blood extraction kit (iNtRON Biotechnology, Seongnam, South Korea). Nucleotide changes were determined by polymerase chain reaction (PCR)-restriction fragment length polymorphism (RFLP) analysis. Restriction enzyme digestion was carried out using the following enzymes (New England BioLabs, Ipswich, MA, USA): *Ban*I (*DICER* rs3742330), *Bcc*I (*DICER* rs13078), *Nla*III (*DROSHA*, rs10719), *Sau*96I (*DROSHA* rs6877842), *Bsl*I (*RAN*, rs14035), and *Bsm*I (*XPO5*, rs11077). Digestion was carried out at 37°C for 16 h.

Genotypes determined by RFLP analysis were confirmed by two independent investigators and by sequencing 10% of the samples.

### Statistical analysis

Data are expressed as the mean ± standard deviation, or n (%) as appropriate. Differences among continuous and categorical variables were examined for statistical significance using the Student’s *t*-test (or the Mann-Whitney test) and the chi-squared test (or Fisher’s exact test). Allele frequencies were calculated to identify deviations from Hardy-Weinberg equilibrium (HWE). Adjusted odds ratios (AORs), hazard ratios (HRs), and 95% confidence intervals (CIs) were used to examine the association between *DICER*, *DROSHA*, *RAN*, *XPO5* polymorphisms and HCC development using GraphPad Prism 4.0 (GraphPad Software Inc., San Diego, CA, USA) and MedCalc version 11.1.1.0 (Medcalc Software, Mariakerke, Belgium).

Survival time was calculated from the date of HCC diagnosis to the date of death or last follow-up (maximum, 60 months). Survival analysis was estimated using Cox proportional hazards regression model. Statistical significance was considered at a level of *P*<0.05.

## Results

### Patient characteristics

The clinical characteristics of the HCC patients and control subjects are shown in Table 1. HCC patients and controls were matched for age and sex (*P* = 0.720 and 0.271, respectively). The proportion of hypertension was significantly lower in patients with HCC than in controls (10.9% vs. 21.1%, *P* = 0.045). The proportion of smoking (51.7% vs. 32.1%) and drinking (57.8% vs. 37.3%) were significantly higher in HCC patients than in control subjects (all *P*<0.05). There were no significant differences in the proportion of patients with diabetes mellitus or in body mass index (all *P*>0.05). Of the HCC patients, 12 (8.2%) underwent surgical resections. The TNM stage distribution was as follows: stage I, 33 (22.4%); stage II, 35 (23.8%); stage III, 47 (32.0%); and stage IV, 32 (27.8%).

**Table 1:** Baseline characteristics of HCC patients and control subjects.

Characteristic	Controls (n = 229)	HCC patients (n = 147)	*P* value
Age (years)	55.28 ± 11.15	55.70 ± 10.94	0.720
Male gender	133 (63.6)	114 (77.6)	0.271
Hypertension	44 (21.1)	16 (10.9)	0.045
Diabetes mellitus	23 (11.0)	26 (17.7)	0.159
Body mass index >25kg/m^2^	43 (20.6)	35 (23.8)	0.650
Smoking	67 (32.1)	76 (51.7)	0.021
Drinking	78 (37.3)	85 (57.8)	0.027
Tumor size			
< 5 cm	-	64 (43.5)	-
≥ 5 cm	-	83 (56.5)	-
Portal vein thrombosis			
No	-	86 (58.5)	-
Yes	-	61 (41.4)	-
Surgical resection			
No	-	134 (91.2)	-
Yes	-	12 (8.2)	-
Chemotherapy/Radiotherapy			
No	-	26 (17.7)	-
Yes	-	120 (81.6)	-
TNM stage			
I	-	33 (22.4)	-
II	-	35 (23.8)	-
III	-	47 (32.0)	-
IV	-	32 (27.8)	-
Child-Pugh class			
A	-	79 (53.7)	-
B	-	32 (21.8)	-
C	-	36 (24.5)	-

HCC, hepatocellular carcinoma.

### Genotype frequencies of microRNA machinery gene polymorphisms in HCC patients and control subjects

Genotype and allele frequencies for the six polymorphisms in the microRNA machinery genes evaluated are shown in Table 2. Genotype distributions in both groups displayed no departure from HWE. There was no significant association between HCC development and the analyzed polymorphisms (Table 2).

**Table 2:** Genotype frequencies of microRNA machinery gene polymorphisms in HCC patients and control subjects.

Characteristics	Controls (n = 209)	HCC patients (n = 147)	AOR (95% CI)*	*P* value
***DICER* rs3742330T>C**				
AA	71 (34.0)	42 (28.6)	1.000 (reference)	
AG	96 (45.9)	82 (55.8)	1.417 (0.813–2.470)	0.219
GG	42 (20.1)	23 (15.6)	0.956 (0.462–1.978)	0.903
Dominant (AA vs. AG + GG)			1.270 (0.752–2.146)	0.371
Recessive (AA + AG vs. GG)			0.722 (0.392–1.329)	0.295
HWE*-P*	0.360	0.103		
***DICER* rs13078A>T**				
AA	192 (91.9)	132 (89.8)	1.000 (reference)	
AT	17 (8.1)	15 (10.2)	1.324 (0.570–3.080)	0.514
TT	0 (0.0)	0 (0.0)	N/A	
Dominant (AA vs. AT + TT)			1.324 (0.570–3.080)	0.514
Recessive (AA + AT vs. TT)			N/A	
HWE*-P*	0.540	0.514		
***DROSHA* rs10719T>C**				
TT	110 (52.6)	81 (55.1)	1.000 (reference)	
TC	88 (42.1)	53 (36.1)	0.924 (0.557–1.532)	0.758
CC	11 (5.3)	13 (8.8)	1.768 (0.651–4.804)	0.264
Dominant (TT vs. TC + CC)			1.033 (0.640–1.668)	0.895
Recessive (TT + TC vs. CC)			1.869 (0.708–4.939)	0.207
HWE*-P*	0.215	0.317		
***DROSHA* rs6877842C>G**				
CC	200 (95.7)	138 (93.9)	1.000 (reference)	
CG	9 (4.3)	9 (6.1)	2.149 (0.672–6.871)	0.197
GG	0 (0.0)	0 (0.0)	N/A	
Dominant (CC vs. CG + GG)			2.149 (0.672–6.871)	0.197
Recessive (CC + CG vs. GG)			N/A	
HWE*-P*	0.750	0.702		
***RAN* rs14035C>T**		0.330143541		
CC	137 (65.6)	98 (66.7)	1.000 (reference)	
CT	69 (33.0)	42 (28.6)	0.968 (0.574–1.633)	0.903
TT	3 (1.4)	7 (4.8)	3.244 (0.609–17.266)	0.168
Dominant (CC vs. CT + TT)			1.072 (0.646–1.779)	0.787
Recessive (CC + CT vs. TT)			3.468 (0.674–17.850)	0.137
HWE*-P*	0.078	0.373		
***XPO5* rs11077A>C**				
AA	170 (81.3)	128 (87.1)	1.000 (reference)	
AC	38 (18.2)	19 (12.9)	0.671 (0.346–1.302)	0.238
CC	1 (0.5)	0 (0.0)	N/A	0.994
Dominant (AA vs. AC + CC)			0.659 (0.341–1.276)	0.216
Recessive (AA + AC vs. CC)			N/A	0.994
HWE*-P*	0.465	0.402		

* The AOR on the basis of risk factors, such as age, gender, hypertension, diabetes mellitus, drinking status, and smoking.

HCC, hepatocellular carcinoma; AOR, adjusted odds ratio; CI, confidence interval.

### Haplotype frequencies of microRNA machinery gene polymorphisms

We evaluated haplotype frequencies to further evaluate the association of microRNA machinery genes with HCC. The haplotype frequencies of microRNA machinery gene polymorphisms in HCC patients and controls are shown in Table 3. Among the models for six polymorphic loci, *DICER* (rs3742330 and rs13078), *DROSHA* (rs10719 and rs6877842), *RAN* (rs14035) and *XPO5* (rs11077), one allele combination (A-A-T-C-C-C) showed synergistic effects in terms of an increased risk of HCC development (OR = 8.881, 95% CI = 1.889–41.750, *P* = 0.002) (Table 3). After adjusting for other risk factors, no allele combination was a significant risk factor for developing HCC (S1 Table).

**Table 3:** Allele combination analysis of microRNA machinery gene polymorphism in HCC patients and control subjects.

Allele combination	Controls (n = 209)	Cases (n = 147)	OR (95% CI)	*P*	FDR
*DICER* rs3742330/*DICER* rs13078/*DROSHA* rs10719/*DROSHA* rs6877842/*RAN* rs14035/*XPO5* rs11077					
A-A-T-C-C-A	0.286	0.229	1.000 (reference)		
A-A-T-C-C-C	0.005	0.034	8.881 (1.889–41.750)	0.002	0.042
A-A-T-G-C-A	0.011	0.022	2.131 (0.627–7.250)	0.335	0.631
A-T-T-C-C-A	0.008	0.016	2.960 (0.686–12.780)	0.150	0.488
A-T-T-G-C-A	0.002	0.000	0.590 (0.024–14.700)	1.000	1.000
A-A-T-C-T-A	0.066	0.089	1.710 (0.924–3.168)	0.110	0.488
A-A-T-C-T-C	0.005	0.000	0.354 (0.017–7.489)	0.539	0.719
A-T-T-C-T-A	0.003	0.010	5.328 (0.543–52.270)	0.142	0.488
A-A-C-C-C-A	0.107	0.126	1.460 (0.861–2.476)	0.175	0.506
A-A-C-C-C-C	0.016	0.000	0.118 (0.007–2.100)	0.098	0.488
A-T-C-C-C-A	0.020	0.016	1.110 (0.349–3.531)	1.000	1.000
A-T-C-G-C-C	0.000	0.003	5.311 (0.213–132.300)	0.364	0.631
A-A-C-C-T-A	0.027	0.015	0.646 (0.198–2.109)	0.581	0.719
A-A-C-C-T-C	0.011	0.000	0.197 (0.010–3.712)	0.299	0.631
A-T-C-C-T-A	0.004	0.006	3.552 (0.316–39.930)	0.555	0.719
G-A-T-C-C-A	0.280	0.278	1.245 (0.825–1.879)	0.346	0.631
G-A-T-C-C-C	0.024	0.000	0.084 (0.005–1.462)	0.017	0.221
G-A-T-G-C-C	0.002	0.000	0.590 (0.024–14.700)	1.000	1.000
G-A-T-C-T-A	0.028	0.039	1.628 (0.681–3.892)	0.361	0.631
G-A-T-C-T-C	0.012	0.010	1.066 (0.247–4.601)	1.000	1.000
G-A-T-G-T-A	0.003	0.006	3.552 (0.316–39.930)	0.555	0.719
G-T-T-C-T-C	0.004	0.000	0.354 (0.017–7.489)	0.539	0.719
G-A-C-C-C-A	0.042	0.069	1.973 (0.976–3.989)	0.068	0.488
G-A-C-C-C-C	0.008	0.018	2.960 (0.686–12.780)	0.150	0.488
G-A-C-C-T-A	0.015	0.016	1.480 (0.435–5.035)	0.535	0.719
G-A-C-C-T-C	0.010	0.000	0.197 (0.010–3.712)	0.299	0.631
G-A-C-G-T-A	0.003	0.000	0.590 (0.024–14.700)	1.000	1.000

HCC, hepatocellular carcinoma; OR, odds ratio; CI, confidence interval; FDR: false positive discovery rate

### MicroRNA machinery genes polymorphisms and survival of HCC patients

**Fig 1:**
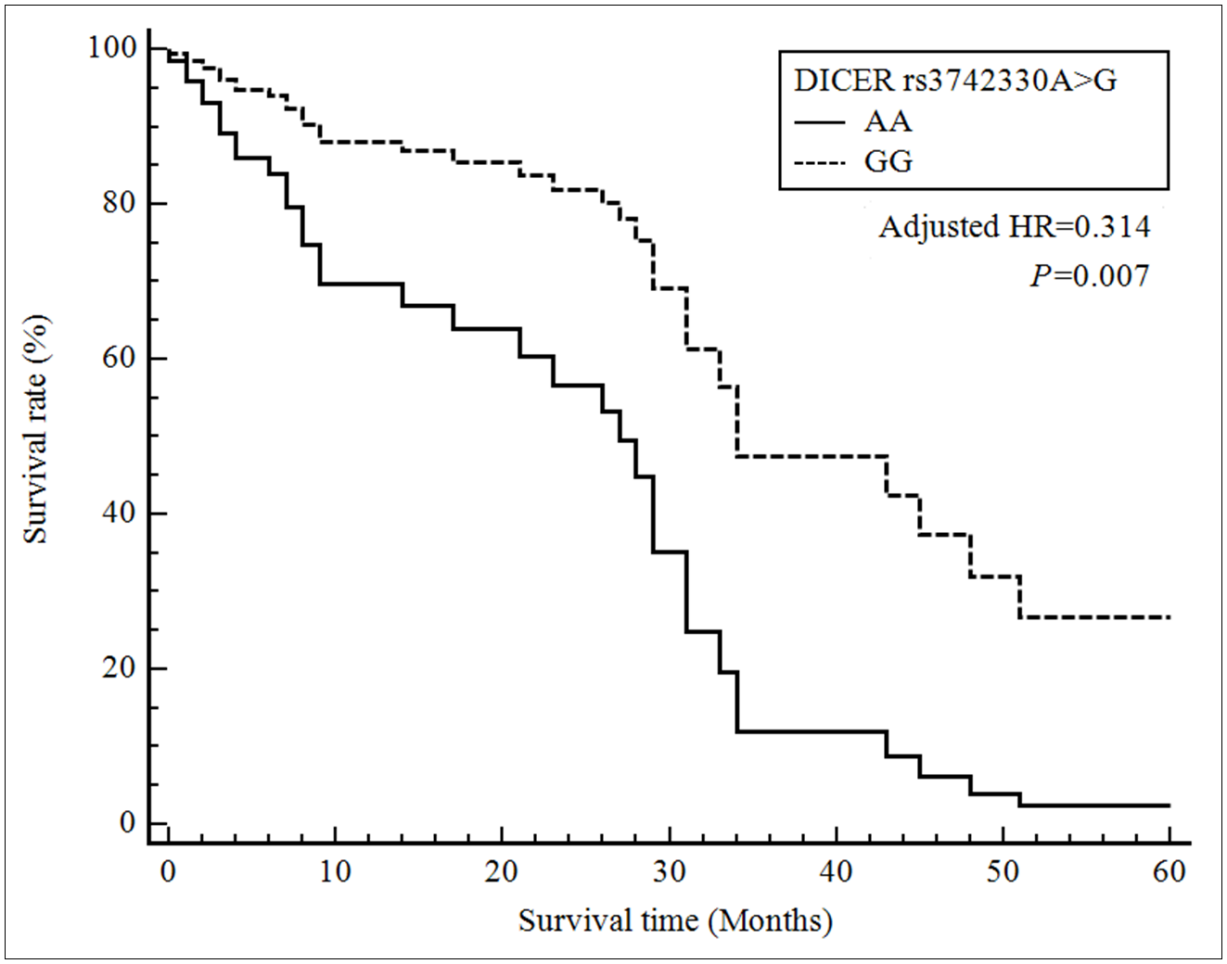
Survival curves for HCC patients with the *DICER* rs3742330 AA genotype (reference) and the GG genotype. Patients carrying the rs3742330 GG genotype had a decreased risk (HR, 0.314; 95% CI, 0.135–0.730; *P* = 0.007) of death compared with those with the AA genotype.

**Fig 2:**
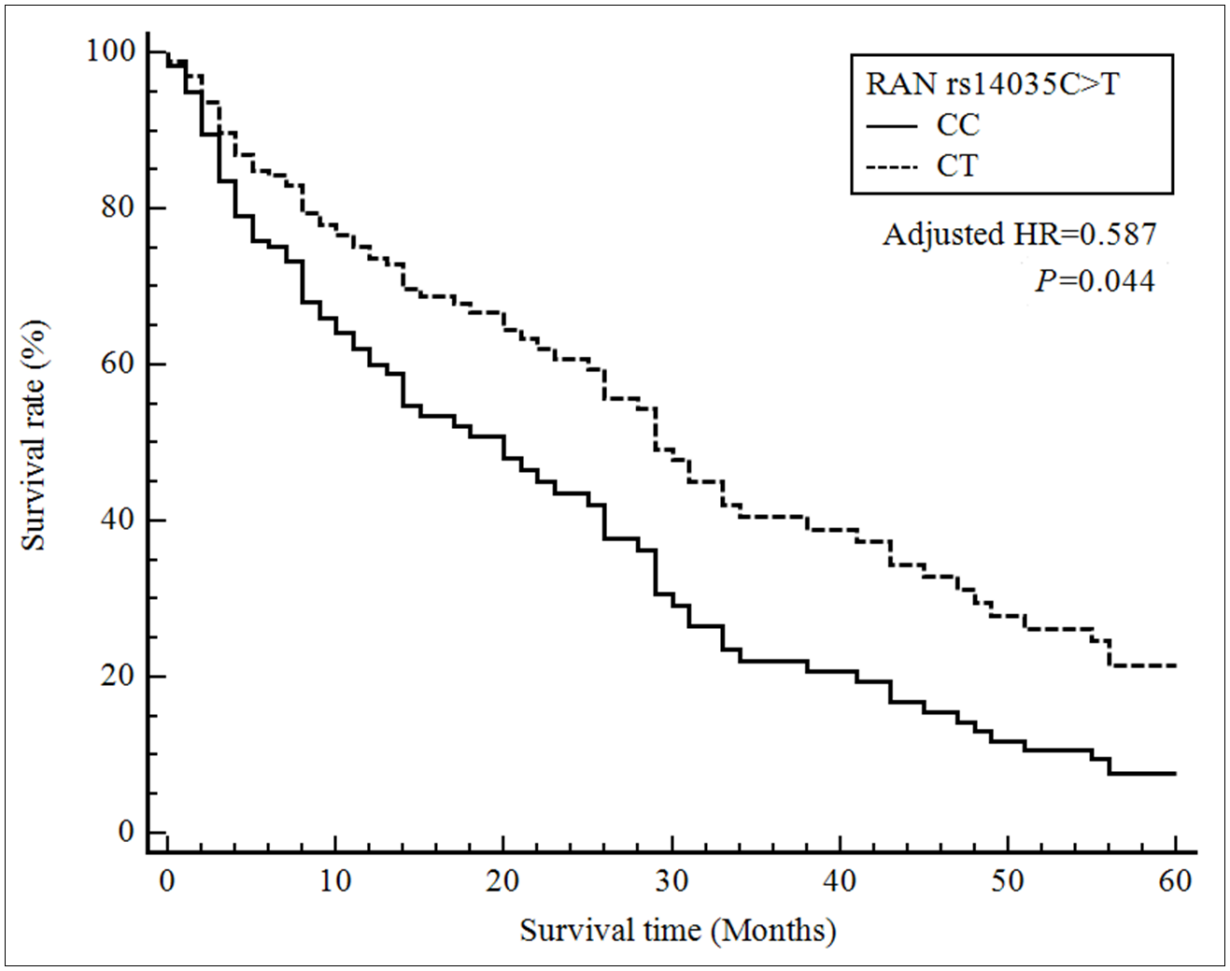
Survival curves for HCC patients with the *RAN* rs14035 CC genotype (reference) and the CT genotype. Patients carrying the *RAN* rs14035 CT genotype showed a decreased risk of death compared with those with the CC genotype (HR, 0.587; 95% CI, 0.349–0.987; *P* = 0.044).

Multivariate Cox proportional hazard regression analysis showed a significant survival benefit for the *DICER* rs3742330 GG genotype compared with the AA genotype (HR = 0.314, 95% CI = 0.135–0.730, *P* = 0.007) and for the *RAN* rs14035 CT genotype compared with the CC genotype (HR = 0.587, 95% CI = 0.349–0.987, *P* = 0.044) (Table 4, Figs 1 and 2). Also, the combined CT + TT genotype was associated with decreased HCC survival (HR = 0.610, 95% CI = 0.380–0.978, *P* = 0.041). The factors affecting survival from the univariate and multivariate Cox-regression analyses were shown in Table 5. Survival of patients with HCC was associated with TNM stage, Child-Pugh class, portal vein thrombosis, surgical resection, chemotherapy or radiotherapy, *DICER* rs3742330 GG genotype, and *RAN* rs14035 CT genotype.

**Table 4:** Genotype frequencies of microRNA machinery genes polymorphism and HCC patients survival based on cox-regression analysis.

Variable	HCC patients (n = 146)	Death (n = 111)	Adjusted hazard ratio* (95% CI)	*P* value
***DICER* rs3742330A>G**				
AA	42 (28.8)	32 (28.8)	1.000(reference)	
AG	81 (55.5)	64 (57.7)	0.749 (0.450–1.248)	0.268
GG	23 (15.8)	15 (13.5)	0.314 (0.135–0.730)	0.007
Dominant (AA vs. AG + GG)			0.681 (0.422–1.099)	0.115
Recessive (AA + AG vs. GG)			0.579 (0.299–1.121)	0.105
***DICER* rs13078A>T**				
AA	131 (89.7)	98 (88.3)	1.000(reference)	
AT	15 (10.3)	13 (11.7)	1.283 (0.658–2.502)	0.464
TT	0 (0.0)	0 (0.0)	N/A	
Dominant (AA vs. AT + TT)			1.283 (0.658–2.502)	0.464
Recessive (AA + AT vs. TT)			N/A	
***DROSHA* rs10719T>C**				
TT	81 (55.5)	62 (55.9)	1.000(reference)	
TC	52 (35.6)	37 (33.3)	0.932 (0.581–1.494)	0.769
CC	13 (8.9)	12 (10.8)	1.061 (0.499–2.256)	0.879
Dominant (TT vs. TC + CC)			0.936 (0.612–1.431)	0.758
Recessive (TT + TC vs. CC)			0.871 (0.444–1.708)	0.687
***DROSHA* rs6877842C>G**				
CC	137 (93.8)	106 (95.5)	1.000(reference)	
CG	9 (6.2)	5 (4.5)	0.730 (0.268–1.983)	0.537
GG	0 (0.0)	0 (0.0)	N/A	
Dominant (CC vs. CG + GG)			0.730 (0.268–1.983)	0.537
Recessive (CC + CG vs. GG)			N/A	
***RAN* rs14035C>T**				
CC	98 (67.1)	77 (69.4)	1.000(reference)	
CT	41 (28.1)	29 (26.1)	0.587 (0.349–0.987)	0.044
TT	7 (4.8)	5 (4.5)	1.283 (0.374–4.175)	0.679
Dominant (CC vs. CT + TT)			0.604 (0.371–0.983)	0.043
Recessive (CC + CT vs. TT)			1.216 (0.401–3.693)	0.730
***XPO5* rs11077A>C**				
AA	127 (87.0)	96 (86.5)	1.000(reference)	
AC	19 (13.0)	15 (13.5)	1.105 (0.594–2.058)	0.752
CC	0 (0.0)	0 (0.0)	N/A	
Dominant (AA vs. AC + CC)			1.105 (0.594–2.058)	0.752
Recessive (AA + AC vs. CC)			N/A	

* Adjusted for age, gender, smoking, drinking, lymph invasion, portal vein thrombosis, tumor size, surgical resection, chemotherapy or radiotherapy, Child-Pugh class and TNM stage

HCC, hepatocellular carcinoma; CI, confidence interval; N/A, non-applicable.

**Table 5:** Results of multivariable Cox-regression analysis of HCC survival.

Covariate	Univariate analysis	Multivariate analysis		
	*P* value	HR	95% CI	*P* value
Tumor stage (TNM, I/II vs. III/IV)	<0.0001	2.986	1.631–5.469	0.000
Child-Pugh class (A vs. B+C)	0.001	1.037	0.142–0.800	0.800
Portal vein thrombosis (Yes vs. No)	<0.0001	2.218	1.267–3.884	0.016
Surgical resection (Yes vs. No)	0.003	0.160	0.050–0.517	0.003
Chemotherapy or Radiotherapy (Yes vs. No)	0.000	0.478	0.280–0.816	0.007
*DICER* rs3742330 GG	0.032	0.688	0.493–0.960	0.028
*RAN* rs14035 CT	0.190	0.648	0.435–0.965	0.033

HCC, hepatocellular carcinoma; HR, hazard ration, CI, confidence interval.

## Discussion

We performed this case-control study to evaluate the associations of *DICER* (rs3742330 and rs13078), *DROSHA* (rs10719 and rs6877842), *RAN* (rs14035) and *XPO5* (rs11077) polymorphisms with HCC. In addition, we evaluated the impact of these microRNA SNPs on survival of HCC patients. To our knowledge, this is the first study to investigate the associations of these six polymorphisms with the risk and prognosis of HCC.

Previous studies have shown that microRNA machinery genes—such as *DICER*, *DROSHA*, *XPO5*, and *RAN*—are associated with cancer development. [17–19] However, in the present study, *DICER* (rs3742330 and rs13078), *DROSHA* (rs10719 and rs6877842), *RAN* (rs14035) and *XPO5* (rs11077) polymorphisms were not associated with the risk of HCC.

In the present study, the microRNA SNPs in *Dicer* and *RAN* were associated with the prognosis of HCC. To our knowledge, this is the first study to provide evidence that *Dicer* and *RAN* are associated with the survival of HCC patients. We report significant associations of the *Dicer* rs3742330 and *RAN* rs14035 SNPs with the survival of HCC patients. Furthermore, a stepwise Cox regression analysis indicated that the *Dicer* rs3742330 and *RAN* rs14035 genotypes, together with TNM stage, portal vein thrombosis, history of surgical resection, and history of chemotherapy or radiotherapy, are independent prognostic factors for survival. Patients carrying the *DICER* rs3742330 GG genotype had a decreased risk (HR, 0.314; 95% CI, 0.135–0.730; *P* = 0.007) of death compared with those with the AA genotype. Also, patients carrying the *RAN* rs14035 CT genotype showed a decreased risk of death compared with those with the CC genotype (HR, 0.587; 95% CI, 0.349–0.987; *P* = 0.044). Identifying this significant prognostic factor may facilitate predicting of those individual patient with better outcomes after diagnosis.

Dicer has been implicated in the oncogenic process of several cancers, but the data are controversial. Down-regulated Dicer expression has been shown in HCC [20], lung cancer [12], ovarian cancer [21], nasopharyngeal cancers [22], breast cancer [23], and esophageal cancer [24], whereas up-regulated Dicer expression was identified in lung adenocarcinoma [13], colorectal cancer [25], and primary cutaneous T-cell lymphomas [26].

Recent reports demonstrated that reduced expression of the Dicer gene was associated with clinical aggressiveness or poorer prognosis for various tumors arising, including the lung and ovary [12,21]. Also, decreased Dicer expression in cancer conferred increased proliferative ability and an invasive phenotype [27,28]. Altered DICER expression may affect microRNAs as a whole, leading to suppression of microRNA expression profiles, thereby influencing the cancer prognosis. The SNP rs3742330 is located in the 3’-untranslated region (UTR) of Dicer, which is a region that might influence the stability and expression of the gene. Recent report demonstrated that the *Dicer* rs3742330 GG genotype was associated with increased overall survival (Variation in Dicer Gene is associated with increased survival in T-cell lymphoma), which is consistent with our study. The underlying mechanism of how this SNP modifies HCC survival remains unclear; it may affect mRNA stability, which is associated with altered *Dicer* expression. Altered *Dicer* expression may affect the microRNA expression profiles, thus, mediating cancer survival. Nevertheless, the mechanism underlying the effect of this SNP on the survival of HCC patients remains unclear. The role of DICER in HCC and the binding of noncoding RNAs, including microRNAs, at this SNP site to mediate DICER expression warrants further investigation.

RAN is a member of the Ras superfamily of GTPasese and is essential for translocation of pre-microRNAs from the nucleus to the cytoplasm [29]. Ran is overexpressed in some cancer cell lines, which supports its role in cancer development [30,31]. The rs14035 polymorphism in the *RAN* 3’-UTR might alter RNA expression, resulting in initiation of carcinogenesis by modulating the production of mature microRNAs [29]. Recent study demonstrated that *RAN* rs14035 CT heterozygotes and T allele (CT + TT genotypes) had a lower colorectal cancer risk than individuals with other genotypes [32]. Yang et al. [33] reported that the *RAN* rs14035 TT genotype was associated with cumulative effects on adverse clinical outcome of esophageal squamous cell cancer. This result is consistent with our results. Although we first reported that the *RAN* rs14035 CT genotype showed a significant survival benefit compared with the CC genotype, the mechanism underlying the effect of the rs14035 SNP in *RAN* on survival of patients with HCC remains unclear.

In conclusion, although we found no direct association between *DICER* (rs3742330 and rs13078), *DROSHA* (rs10719 and rs6877842), *RAN* (rs14035) and *XPO5* (rs11077) polymorphisms and the risk of HCC, we demonstrated that *DICER* (rs3742330) and *RAN* (rs14035) are associated with the survival of HCC patients. An important limitation of our study was the small sample size, which prevented us from drawing definitive conclusions. Therefore, our results should be interpreted with caution.

To our knowledge, our study is the first to analyze the association between polymorphisms in these six microRNA machinery genes and HCC risk and prognosis. Our results provide a more comprehensive understanding of the relationship between microRNA machinery gene polymorphisms and HCC. However, the results from this study require validation in other populations and laboratory-based functional studies. Future studies using a larger sample size are needed to further evaluate the role of polymorphisms in microRNA machinery genes in the risk and prognosis of HCC.

## Supporting Information

S1 Table Allele combination analysis of microRNA machinery gene polymorphism in HCC patients and control subjects. (DOC)

## Abbreviations

AOR: adjusted odds ratio
CI: confidence interval
HCC: hepatocellular carcinoma
HR: hazard ratio
HWE: hardy-weinberg equilibrium
PCR: polymerase chain reaction
RFLP: restriction fragment length polymorphism
SNP: single nucleotide polymorphism
UTR: untranslated region
