# Supplementary material for: Variation in the Dicer and RAN Genes Are Associated with Survival in Patients with Hepatocellular Carcinoma
Source: PLoS One. 2016 Sep 9;11(9):e0162279. doi: 10.1371/journal.pone.0162279 (PMC5017754; doi:10.1371/journal.pone.0162279)
Supplement: S1 Table — (DOC) [file pone.0162279.s001.doc]

**S1 Table.** **Allele combination analysis of microRNA machinery gene polymorphism in HCC patients and control subjects**

| **Allele combination** | **Controls (n=209)** | **Cases (n=147)** | **AOR (95% CI)*** | ***P*** | **FDR** |
| --- | --- | --- | --- | --- | --- |
| *DICER* rs3742330/*DICER* rs13078/*DROSHA* rs10719/*DROSHA* rs6877842/*RAN* rs14035/*XPO5* rs11077 | | |  |  |  |
| A-A-T-C-C-A | 0.286 | 0.229 | 1.000 (reference) |  |  |
| A-A-T-C-C-C | 0.005 | 0.034 | 4.311 (0.415-44.790) | 0.221 | 0.884 |
| A-A-T-G-C-A | 0.011 | 0.022 | N/A | 0.998 | 0.998 |
| A-T-T-C-C-A | 0.008 | 0.016 | 1.453 (0.124-17.057) | 0.766 | 0.998 |
| A-T-T-G-C-A | 0.002 | 0.000 | N/A | 0.998 | 0.998 |
| A-A-T-C-T-A | 0.066 | 0.089 | 2.312 (0.742-7.210) | 0.149 | 0.884 |
| A-A-T-C-T-C | 0.005 | 0.000 | N/A |  |  |
| A-T-T-C-T-A | 0.003 | 0.010 | 1.673 (0.145-19.342) | 0.680 | 0.998 |
| A-A-C-C-C-A | 0.107 | 0.126 | 1.330 (0.669-2.642) | 0.416 | 0.929 |
| A-A-C-C-C-C | 0.016 | 0.000 | 0.601 (0.045-8.009) | 0.700 | 0.998 |
| A-T-C-C-C-A | 0.020 | 0.016 | 2.557 (0.253-25.810) | 0.426 | 0.929 |
| A-T-C-G-C-C | 0.000 | 0.003 | N/A | 0.998 | 0.998 |
| A-A-C-C-T-A | 0.027 | 0.015 | 0.479 (0.129-1.773) | 0.270 | 0.924 |
| A-A-C-C-T-C | 0.011 | 0.000 | N/A | 0.998 | 0.998 |
| A-T-C-C-T-A | 0.004 | 0.006 | 4.179 (0.268-65.246) | 0.308 | 0.924 |
| G-A-T-C-C-A | 0.280 | 0.278 | 0.825 (0.521-1.307) | 0.412 | 0.929 |
| G-A-T-C-C-C | 0.024 | 0.000 | 0.902 (0.223-3.654) | 0.885 | 0.998 |
| G-A-T-G-C-C | 0.002 | 0.000 | N/A |  |  |
| G-A-T-C-T-A | 0.028 | 0.039 | 1.792 (0.808-3.976) | 0.151 | 0.884 |
| G-A-T-C-T-C | 0.012 | 0.010 | 0.649 (0.099-4.247) | 0.651 | 0.998 |
| G-A-T-G-T-A | 0.003 | 0.006 | N/A | 0.998 | 0.998 |
| G-T-T-C-T-C | 0.004 | 0.000 | N/A | 0.998 | 0.998 |
| G-A-C-C-C-A | 0.042 | 0.069 | 1.569 (0.770-3.195) | 0.215 | 0.884 |
| G-A-C-C-C-C | 0.008 | 0.018 | 1.589 (0.368-6.857) | 0.535 | 0.998 |
| G-A-C-C-T-A | 0.015 | 0.016 | 1.994 (0.683-5.815) | 0.207 | 0.884 |
| G-A-C-C-T-C | 0.010 | 0.000 | 0.247 (0.049-1.242) | 0.090 | 0.884 |
| G-A-C-G-T-A | 0.003 | 0.000 | 1.533 (0.087-27.177) | 0.771 | 0.998 |
| HCC, hepatocellular carcinoma; AOR, adjusted odds ratio; CI, confidence interval; FDR: false positive discovery rate. | | | | | |
| *Adjusted for age, gender, smoking, drinking, hypertension, and diabetes mellitus. |  |  |  |  |  |
